# Drug therapy problems, medication adherence and treatment satisfaction among diabetic patients on follow-up care at Tikur Anbessa Specialized Hospital, Addis Ababa, Ethiopia

**DOI:** 10.1371/journal.pone.0222985

**Published:** 2019-10-01

**Authors:** Gebre Teklemariam Demoz, Alemseged Beyene Berha, Minyahil Alebachew Woldu, Helen Yifter, Workineh Shibeshi, Ephrem Engidawork

**Affiliations:** 1 Department of Clinical Pharmacy, School of Pharmacy, College of Health Sciences, Aksum University, Aksum, Ethiopia; 2 Department of Pharmacology and Clinical Pharmacy, School of Pharmacy, College of Health Sciences, Addis Ababa University, Addis Ababa, Ethiopia; 3 Department of Internal Medicine, School of Medicine, College of Health, Sciences Addis Ababa University, Addis Ababa, Ethiopia; University of Toronto, Rotman School, CANADA

## Abstract

**Background:**

Patients with diabetes are at high risk of drug therapy problems (DTPs), as they are receiving multiple medications. To date, studies regarding DTPs in patients with diabetes in Ethiopia are limited. The aim of this study was to assess prevalence of DTPs, medication adherence and treatment satisfaction of patients with diabetes at Tikur Anbessa Specialized Hospital (TASH).

**Method:**

A cross-sectional study was conducted on randomly selected 418 participants who fulfilled the inclusion criteria. Data were collected using structured questionnaire and patients’ chart review. Cipolle’s classification system was used to determine DTPs. Modified Morisky’s Adherence Scale (MMAS-8) was used to measure patients’ adherence to their medication. Treatment Satisfaction with Medicines Questionnaire (SATMED-Q) patient satisfaction assessment questionnaire was used to assess patients’ treatment satisfaction.

**Results:**

A total of 207 DTPs in 177 (42.3%) of participants were identified. Commonly identified DTPs were dosage too low (58, 28.0%), ineffective drug therapy (54, 26.1%), and need additional drug therapy (52, 25.1%). Factors associated with DTPs were female gender (Adjusted Odds Ratio [AOR] = 2.31,95% CI:1.30–4.12); ≥3comorbidities (AOR = 3.61, 95% CI:1.19–10.96); ever married (AOR = 2.58,95% CI:1.23–5.48); type 2 diabetes (AOR = 5.62, 95% CI:1.21–26.04); non-adherence (AOR = 5.26,95% CI:2.51–11.04) and residence out of Addis Ababa (AOR = 0.30, 95% CI:0.12–0.73). Twenty four percent of participants were non-adherent to their drug therapies. Factors associated with non-adherence were diabetes complications (AOR = 2.00, 95% CI: 1.2–3.32), the female gender (AOR = 1.67, 95%CI: 1.01–2.8) and level of education (AOR = 0.42, 95%CI: 0.18–0.96). Eighty one percent of participants were satisfied with the current treatment.

**Conclusion:**

A significant proportion of patients were satisfied with their treatment and a quarter of the study participants were non-adherent to their medications at TASH diabetic clinic. However, DTPs were considerably higher among the study participants. Hence, future interventions targeting prevention and resolution of DTPs deemed to be necessary.

## Introduction

Diabetes Mellitus (DM, diabetes) is a group of metabolic diseases characterized by the occurrence of persistent hyperglycemia due to deficiency in insulin secretion, insulin action or both. DM can be classified as type 1, type 2, gestational and other types of diabetes [1–3]. In the most recent World Health Organization (WHO) global report, a dramatic increase of diabetes is largely due to the rise in Type 2 Diabetes (T2DM) [4].

Globally, diabetes is the third among the five leading global risks for mortality [5]. According to the International Diabetes Federation (IDF), about 425 million (8.8%) adults were living with diabetes worldwide in 2017 and this is projected to increase to 629 million (9.9%) by 2045, out of which about 108 million would be in the Africa region [3]. In Ethiopia, about 1.8 million adults were living with diabetes [4,6]. The WHO reports indicated that the prevalence of diabetes in Ethiopia increased from 0.5% in 1980 through to 3.8% in 2014 [6] to 5.2% in 2017 [3]

Drug Therapy Problems (DTPs) are events or circumstances involving or suspected to be involved during drug therapy that actually or potentially interfere with a desired health outcomes [7,8]. DTPs can be classified into various categories in the literature. To date, the most widely used classification system for DTPs is the Cipolle et al [8], and accordingly there are seven distinct categories of DTPs. These are unnecessary drug therapy, need for additional drug therapy, ineffective drug therapy, dosage too low, dosage too high, adverse drug reaction and non-adherence [8]. Patients with diabetes are, especially, at high risk for experiencing DTPs. Studies reported that most diabetes patients had at least one DTP [9–12]. This is because patients with diabetes are subject to receive multiple drug therapies for their multiple co-morbidities associated with DM [9,13]. The occurrences of DTPs are very frequent especially among ambulatory patients [9,13,14]. Hence, identifying a drug therapy problem is a clinical judgment that requires the practitioner to identify an association between the patient’s medical condition and the patient’s pharmacotherapy [8].

Medication adherence is also one of the most important factors that determine therapeutic outcomes, especially in patients with DM. Whatever the efficacy of a drug, it won’t be working unless the patient takes it as recommended by the Health Care Professionals (HCP)[15]. The term adherence means, “sticking to a plan” for perceived benefit. This concept is applied for patients, who have adopted and integrated a plan given by the HCP. Adherence with medication regimens is essential for attaining maximal therapeutic benefits [16]. Poor adherence to prescribed medications has been identified as a barrier in controlling chronic illnesses including DM. When patients do not take medications as prescribed they suffer with severe consequences that lead to poor quality of life [17]. Non-adherence is considered a key predictor for the failure of patients to attain and maintain their treatment goals, which associates with poor health and quality of life outcomes [18]. According to WHO report, 50% of patients from developed countries with chronic diseases do not use their medications as recommended. In DM, adherence rates are particularly problematic, generally ranging from 30% to 70% [19].

Treatment satisfaction is an important factor of quality of care, especially in treating chronic diseases such as diabetes mellitus. Assessment will be helpful for identifying factors that independently influence treatment satisfaction by improving clinical outcomes [20]. Furthermore, treatment satisfaction may be important for differentiating among diabetes treatments, and for monitoring patient outcomes in clinical practice [21]. Therefore, the primary aims of this study were to determine the prevalence of DTP, medication adherence and treatment satisfaction among patients with diabetes at Tikur Anbessa Specialized Hospital (TASH).

## Methods

### Study setting

The study was conducted at TASH. TASH is the largest public referral and teaching hospital under Addis Ababa University (AAU), Ethiopia. TASH is the training center for postgraduate and undergraduate health science students. The hospital has about 800 beds and provides all round treatment services for approximately 370,000–400,000 patients coming from the different parts of the country per year [22]. The hospital has a number of outpatient follow up clinics for chronic illnesses. Adult patients with T1DM and T2DM are scheduled to visit the diabetic clinic every morning on Mondays and Wednesday. About 230 diabetes patients per week get service in the clinic.

### Study design and population

This hospital based cross sectional study was carried out from 1 April to 30 June 2017 and data spanning from June 2016 to June 2017 were extracted from patient charts. All adult diabetic patients who visited the clinic during the study period formed the source population, while patients fulfilling the selection criteria were considered as the study population.

#### Inclusion and exclusion criteria

Patients greater than 18 years old and who had been on follow-up for at least the last 6 months in the clinic were included in the study. Severely ill patients requiring urgent medical care and unwilling persons to participate in the study were excluded.

### Sample size determination and sampling technique

The sample size required was calculated using a single proportion sample size estimating formula.

$$n={\left(Z\alpha /2\right)}^{2}P\frac{\left(1-P\right)}{d^{2}}$$

(Where n = required initial sample size, *Z a*/2 = critical value for normal distribution at 95% confidence interval which equals 1.96 (*Z* value at alpha = 0.05), *P* = proportion of diabetic population having DTPs; (p = 0.5), *q* = proportion of diabetic population not having DTPs (*q* = 0.5), and *d* = marginal error (5% = 0.05)) [19].

Calculation provided ‘n’ value of 384. Considering 10% contingency (for non-response rate), the final sample size of the study became 423.

### Data collection instruments and procedures

Primary and secondary data were collected using structured data collection tools, which included questionnaire and data abstraction format, respectively. The primary data, such as patients’ socio-demographics, clinical data (including reported drug adverse events), medication adherence and reasons for poor adherence were collected through interviewer administered questionnaire. Modified Morisky’s Adherence Scale (MMAS) free online version [23] was used to measure patients’ adherence to their medication. MMAS is an 8-item self-report measure of adherence. Items 1 through 7 have response choices “yes” or “no”, whereas item 8 has a 5-point likert response choices. Each ‘no’ response was rated as ‘1’ and each ‘yes’ as ‘0’ except for item 5 (reversed), in which each response ‘yes’ was rated as ‘1’ and ‘no’ as ‘0’. Item 8 concerning the difficulty to remember taking medications was scored as “Never/Rarely = 0, Once in a while = 1, sometimes = 2, usually = 3 and all the time = 4. The total score range from 0 to 8 and grouped into three levels: high adherence (score = 8), medium adherence (score of 6 to < 8), and low adherence, score< 6.

Regarding DTP assessment, the first two categories were associated with the INDICATION. The third and fourth categories with EFFECTIVENESS, and the fifth and sixth categories with SAFETY. However, the seventh category that dealt with patient ADHERENCE was removed since MMAS was used instead. During reporting each value (frequency) was recorded as prevalence of the specific category and then converted to percentage. Evidence-based medicine is facilitated by these logical DTP categories. The logical and comprehensive categorizations of drug therapy problems facilitate the application of population-based evidence to an individual patient’s problems [8].

Reported ADR data were extracted from patient charts as well as self-reported by patients and interpreted as ADR by attending physicians.

With respect to assessment of treatment satisfaction, the Treatment Satisfaction with Medicines Questionnaire (SATMED-Q) tool was used after receiving permission from the Mapi Research Trust Company via email. The SATMED-Q has demonstrated appropriate psychometric properties, exploring patients’ satisfaction with treatment that will have an impact on treatment outcomes. The SATMED-Q is composed of 17 items investigating six domains exploring actual satisfaction with drug efficacy, side effects, convenience of use, medical care, impact on activities of daily living, and general satisfaction. It also provides a total score for treatment satisfaction with medicines by adding up all domains. Totaling the direct scores of the items yields a total composite score ranging between 0 and 68. The resultant total composite score could be transformed to a more intuitive and easier to understand metric with a minimum of 0 and a maximum of 100, using the following expression [24]:

$$Y^{'}=\frac{Yobs-Ymin}{Ymax-Ymin}x100$$

Where Y_max_ is 68 (maximum total score), Y_min_ is 0 (minimum total score), Y_obs_ is the total patient score, and Y’ is the transformed score. A similar expression can be used to change the metric of each individual domain.

### Data quality management

Pre-test was performed on 5% of the sample one week before the actual data collection and amendment was made accordingly. Training was given to the data collectors and close supervision was performed on daily basis. At the end of each data collection days, completeness of filled questionnaires and recorded information was checked to ensure quality of the data. Immediate correction was made, if any errors were identified.

### Data analysis

Data was entered into Epi Info Version 4.0.2.0 and analyzed using SPSS version 22. Descriptive statistics including mean and standard deviation for continuous variables and frequency and percentage for categorical data were used to summarize socio-demographic and relevant clinical data. Statistical significance was considered at p value≤0.05. Bivariate and multivariate logistic regression analyses were performed to investigate associations of variables with the occurrence of DTPs. Variables were then checked for absence of collinearity and variables with *p*-value ≤ 0.20 in the bivariate analyses were further analyzed in multivariate logistic regression to control the effect of confounders [25,26].

### Ethical considerations

Ethical clearance was received from the Ethical Review Board of School of Pharmacy of AAU prior to data collection and subsequent permission was obtained from the study site department, head of the endocrine and metabolism unit of TASH. Written consent was also obtained from each participant. The right of participants to withdraw from the study at any time or not to participate in the study was respected. Before conducting the interview, the purpose of the study was explained for each participant. Anonymity and confidentiality were maintained through removing identifiers and restricting data access, respectively.

## Results

### Socio-demographic characteristics

These include sex, age, marital status, religion, place of residence, educational status, employment status, smoking status, alcohol consumption and physical activity.

A total number of 418 participants were involved in the study and more than half of them (52.9%) were females. Majority (85.4%) of the participants were patients with T2DM. The mean (±SD) age of participants was 53 ± 13.6 years and nearly half (48.8%) of them were middle aged (41–60 years). Majority (81.0%) of the participants were ever married (at least married once in their lives) and 86.3% of them resided in Addis Ababa city. In this study, 41.7% of the participants attended at least College/University level of education and 57% of the participants were employed. A small proportion of the participants were smokers (0.7%) and more than 70% of the participants stated that they were adherent to regular physical activity (Table 1).

**Table 1:** Socio-demographic characteristics of ambulatory patients with diabetes on follow up at TASH, Addis Ababa, Ethiopia, 2017(n = 418).

Variables	Categories	Study participants (n, %)		
		Type1, n = 61	Type2, n = 357	Total
**Sex**	Male	29(47.5)	168(47.1)	197(47.1)
	Female	32(52.5)	189(52.9)	221(52.9)
**Age (years)**	Mean **±** SD	34.9±10.8	56.1±11.6	53±13.6
	20–40	44(72.1)	22(6.2)	66(15.8)
	41–60	15(24.6)	188(52.7)	203(48.6)
	>60	2(3.3)	147(41.1)	149(35.6)
**Marital status**	Single	28(45.9)	13(3.6)	41(9.8)
	Married	30(49.2)	308(86.2)	338(81.0)
	Divorced	2(3.3)	20(5.6)	22(5.1)
	Widowed	1(1.6)	16(4.5)	17(4.1)
**Religious**	Orthodox	49(80.3)	288(80.7)	337(80.6)
	Muslim	4(6.6)	36(10.1)	40(9.6)
	Protestant	5(8.2)	27(7.6)	32(7.7)
	Others*	3(4.9)	6(9.8)	9(2.1)
**Place of residence**	Addis Ababa	57(93.4)	304(85.2)	316(86.3)
	Out of Addis Ababa	4(6.6)	53(14.8)	57(13.7)
**Educational status**	No formal education	2(3.3)	50(14.0)	52(12.4)
	Primary(1–8)	8(13.1)	63(17.6)	71(17.0)
	Secondary(9–12)	18(29.5)	103(28.9)	121(28.9)
	College/University	33(54.1)	141(39.5)	174(41.7)
**Employment status**	Employed	36(59.1)	201(56.3)	238(56.9)
	Unemployed	25(41.0)	156(43.7)	181(43.3)
**Smoking status**	Current smoker	1(1.6)	2(0.6)	3(0.7)
**Alcohol consumption**	Regular user	4(6.6)	40(11.2)	44(10.5)
**Physical activity**	Regular activity	51(83.6)	252(70.6)	303(72.5)

Others*: Catholic and Seventh day church followers. SD: standard deviation. Percentages are calculated per column.

### Clinical characteristics

These include BMI, hospitalization, average FBG, presence of co-morbid conditions, number of co-morbidities per patient, presence of diabetes complications and number of complications per patient.

The mean duration of diabetes was 11.2 ± 8.9 years. Of the total study participants, 70.6% had co-morbid conditions with a mean of 1.64 ± 0.67 co-morbidities per patient. The most common co-morbidities were hypertension (47.8%), dyslipidemia (42.6%) and ischemic heart disease (8.9%). Moreover, 28.5% participants had developed chronic diabetes complications. The most commonly encountered diabetes complication was diabetic neuropathy (21.5%) followed by diabetic retinopathy (6.5%) and diabetic nephropathy (4.5%). Hospitalization events at least once within the last year were also reported due to acute diabetes complications (29.2%), out of these 23.7% were due to hyperglycemia and 5.5% due to hypoglycemia (Table 2).

**Table 2:** Clinical characteristics of ambulatory patients with diabetes on follow up at TASH, Addis Ababa, Ethiopia, 2017(n = 418).

Variables	Categories	Study participants (n, %)		
		Type 1	Type 2	Total
**Duration of diabetes (years)**	Mean ± SD	12.18±6.66	11.64±6.95	11.2±.89
	1–5	12(19.7)	67(18.8)	79(18.9)
	5–10	19(31.1)	105(18.4)	124(29.7)
	11–15	17(27.9)	95(26.6)	112(26.8)
	>15	13(21.3)	90(25.2)	103(24.6)
**BMI (kg/m**^**2**^**), (n = 77)**	Mean ± SD	23±3.0	27.15±4.46	26.88±5.44
**Hospitalization within last year**	(n,%, yes)	40(65.6)	82(23.0)	122(29.2)
**Access for SMBG**	(n,%, yes)	43(70.5)	274(76.8)	317(75.8)
**Average FBG (mg/dL)**	Mean ± SD	178.40±57.6	172.60±44	172.0±52.0
	<70	3(4.9)	2(0.6)	5(1.2)
	70–130	14(23.0)	62(17.4)	76(18.2)
	>130	44(72.1)	293(82.1)	337(80.6)
**Presence of co-morbid conditions**	(n,%, yes)	17(27.9)	278(77.9)	295(70.6)
	Hypertension	12(19.7)	188(52.7)	200(47.8)
	Dyslipidemia	7(11.5)	171(47.9)	178(42.6)
	IHD	0(0.0)	37(10.4)	37(8.9)
	Others*	4(6.6)	50(14.0)	54(12.9)
**No. of co-morbidities per patient**	Mean ± SD	1.32± 0.6	1.66± 0.66	1.64±0.67
	1–2	17 (27.9)	252(70.6)	269(92.2)
	≥3	0(0.0)	26 (7.3)	26 (8.8)
**Presence of diabetes complications**	(n,%, yes)	4(6.6)	115(32.2)	119(28.5)
	Neuropathy	3(4.9)	87(24.4)	90(21.5)
	Retinopathy	2(3.3)	25(7.0)	27(6.5)
	Nephropathy	1(1.6)	18(5.0)	19(4.5)
**Number of complications per patient**	Mean ± SD	1.50± 0.6	1.22±0.53	1.24±0.52
	1–2	6(9.8)	108(30.3)	114(95.8)
	≥3	0 (0.0)	5 (1.4)	5 (4.2)

Others*: Asthma and Thyroid disorders. FBG: Fasting Blood Glucose, IHD: Ischemic heart disease, SMBG: Self-Monitoring of Blood Glucose.

### Pattern of prescribed medications

Participants had a Mean **±** SD of 3.89 **±**1.87 medications per prescription (each drug that appeared on the prescription was counted regardless of its category or class). Participants with T2DM were either on oral hypoglycemic agents (OHGA) alone (46.8%), OHGA plus insulin (30.8%), or insulin alone (22.4%). In addition, anti-hypertensive medications (48.6%), statins (48.6%), and aspirin (40.7%) were the most frequently prescribed drugs (Table 3).

**Table 3:** Prescribed medications among ambulatory patients with diabetes on follow up at TASH, Addis Ababa, Ethiopia, 2017 (n = 418).

Treatment	Categories	Study participants (n, %)		
		Type 1	Type 2	Total
**Antidiabetic treatment regimen**	OHGA alone	0	167 (46.8)	167 (40.0)
	OHGA + Insulin	0	110 (30.8)	110 (26.3)
	Insulin alone	61 (100)	80(22.4)	141(33.7)
**Specific antidiabetic medications**	Metformin	0	46(12.9)	46(11.0)
	Glibenclamide	0	11(3.1)	11(2.6)
	Metformin + Glibenclamide	0	101(30.8)	101(24.2)
	Metformin +Insulin	0	110(28.4)	110(26.3)
	Metformin + Glimepiride	0	9(2.4)	9(2.2)
**Other medications**	Antihypertensives	12 (2.9)	191(45.7)	203(48.6)
	Angiotensin converting enzyme inhibitors/ARBs	11 (2.6)	166(39.7)	177(42.3)
	Calcium channel blockers	5(1.2)	71(17.0)	76(18.2)
	Beta blockers	1(0.2)	44(10.5)	45(10.8)
	Diuretics	3(0.7)	63(15.1)	66(15.8)
	Antiplatelets(Aspirin)	4(1.0)	166(39.7)	170(40.7)
	Lipid lowering agents(Statins)	7(1.7)	196(46.9)	203(48.6)
	Antiasthmatics	1(0.2)	9(2.2)	10(2.4)
	Others*	7(1.7)	113(26.1)	120(27.8)
**Number of medications per patient**	Mean ± SD	2.15±1.2	4.18±1.8	3.89±1.87
	<5	55 (13.2)	201(48.2)	256 (61.5)
	≥5	5 (1.20)	156 (34.0)	161 (38.5)
**Source of medication (n,%, for free)**	For free	43(70.5)	220(61.6)	263(62.9)
**Use of traditional medicine (n,%, yes)**	Occasional	2(3.3)	11(3.1)	13(3.1)

*Proton pump inhibitors, amitriptyline, carbamazepine, gabapentin, tramadol and proplythiouracil. SD: standard deviation, OHGA: oral hypoglycemic agents, ARBs: Angiotension II receptor blockers

### Drug therapy problems

A total of 207 DTPs were encountered with a mean (±SD) of 1.16 (±0.42) DTPs per patient and majority (93.2%) of them were identified among T2DM patients (Table 4). One or more DTPs were identified in 42.3% of the study participants, of which 84% had only one DTP. The most commonly encountered type of DTP was dosage too low (28.0%) followed by ineffective drug therapy (26.1%) and need additional drug therapy (25.1%). The commonly encountered medications in DTPs were insulin (44.9%), metformin plus insulin (39.6) and metformin plus glibenclamide (26.6%). In addition, lipid lowering agents (25.6%) and aspirin (18.8%) had also contributed to DTPs (See Table 4).

**Table 4:** Drug therapy problems and causes among ambulatory patients with diabetes on follow up at TASH, Addis Ababa, Ethiopia, 2017 (n = 207).

Drug therapy problems and causes	Study participants (n, %)		
	Type 1	Type 2	Total
Dosage too low	11(5.3)	47(22.7)	58(28.0)
Dose too low	11(5.3)	47(22.7)	58(28.0)
Ineffective drug therapy	0(0.0)	54(26.1)	54(26.1)
Inappropriate drug selection	0(0.0)	54(26.1)	54(26.1)
Need additional drug therapy	0(0.0)	52(25.1)	52(25.1)
Synergistic drug therapy	0(0.0)	30(14.5)	30(14.5)
Prophylaxis drug therapy	0(0.0)	16(7.7)	16(7.7)
Untreated medical condition	0(0.0)	6(2.9)	6(2.9)
Unnecessary drug therapy	0(0.0)	20(9.7)	20(9.7)
Duplication drug therapy	0(0.0)	20(9.7)	20(9.7)
Adverse drug reaction	2(1.0)	16(5.8)	18(8.7)
Undesirable drug effect	2(1.0)	12(5.8)	14(6.8)
Unsafe or the patient/contraindication	0(0.0)	4(1.9)	4(1.9)
Dosage too high	1(0.5)	4(1.9)	5(2.4)
Over therapeutic dose	1(0.5)	4(1.9)	5(2.4)
Total number of identified DTPs	14(6.8)	193(93.2)	207(100)
Total number of participants with DTPs	13(3.1)	164(39.2)	177(42.3)
Average number of main DTPs per patient	1.02±0.21	1.2±0.61	1.16±0.42

Percentages are calculated per row, which was per 207 problems. DTPs: drug therapy problems.

#### Factors associated with drug therapy problems

Age, Sex, Marital status, Educational level, Employment status, Place of residence, Physical exercise, Access for Self-Monitoring of Blood Glucose (SMBG), Type of diabetes, Duration of disease, comorbidities, number of co-morbidities, Complication, number complications and type of antidiabetic medications were subjected to bivariate logistic regression analysis. Subsequent multivariate logistic regression analysis revealed T2DM (p = 0.027), presence of greater than or equal to 3 co-morbidities (p = 0.3), being married, being female, poor medication adherence as well as the use of insulin only as antidiabetic treatment (p = 0.34) had significant association with DTPs (Table 5).

**Table 5:** Bivariate and multivariate analysis of factors associated with drug therapy problems among patients with diabetes on follow up at TASH, Addis Ababa, Ethiopia, 2017.

		Drug Therapy Problem		Odds Ratios		Multivariate p-value
Covariates	Categories (n, %)	Yes(n = 177)	No (n = 241)	Crude (95% CI)	Adjusted (95% CI	
Age	Mean(±SD)	53±13.6		1.00(0.99–1.02)		
	20–60	124(41.8)	173(58.2)	1.00		
	60+	68(43.8)	124(41.8)	1.09(0.71–1.67)		
Sex	Male	70(35.2)	127(64.8)	1.00		
	Female	107(48.4)	114(51.6)	1.70(1.15–2.52)**	2.31(1.30–4.12)	**0.004**
Marital status	Never married	10(24.40)	31(75.60)	1.00		
	Ever married	167(44.3)	210(55.7)	2.47(1.18–5.17)**	2.58(1.23–5.48)	**0.013**
Educational status	No formal	24(46.2)	28(53.8)	1.19(0.64–2.21)		
	Primary(1–8)	26(36.6)	45(63.4)	0.80(0.45–1.41)		
	Secondary(9–12)	54(44.6)	67(55.4)	1.12(0.70–1.78)		
	College/University	73(42.0)	101(58.0)	1.00		
Employment status	Employed	97(40.9)	140(59.1)	1.00		
	Unemployed	80(44.2)	101(55.8)	1.14(0.77–1.69)		
Place of residence	Addis Ababa	162(44.9)	199(55.1)	1.00		
	Out of Addis	15(26.3)	42(73.7)	0.44(0.24–0.82)**	0.30(0.12–0.73)	**0.008**
Physical exercise	No	55(47.8)	60(52.2)	1.36(0.88–2.09)*	0.91(0.50–1.65)	0.76
	Yes	122(40.3)	181(59.7)	1.00		
Access for SMBG	No	44(43.6)	57(56.4)	1.07(0.68–1.68)		
	Yes	133(42.0)	184(58.0)	1.00		
Type of diabetes	Type 1	13(21.3)	48(78.7)	1.00		
	Type 2	164(45.9)	193(54.1)	3.14(1.64–6.00)**	5.62(1.21–26.04)	**0.027**
Duration of disease	Mean(±SD)	11.2 ± 8.9		1.01(0.98–1.04)		
	1–5	29(36.7)	50(63.3)	1.00		
	6–10	56(45.2)	68(54.8)	1.42(0.80–2.53)		
	>10	92(42.8)	123(57.2)	1.29(0.76–2.19)		
Comorbidities	No	78(36.6)	115(63.4)	1.00		
	Yes	132(44.7)	163(55.3)	1.40(0.91–2.16)*	1.08(0.59–1.94)	0.26
Complication	No	118(39.5)	181(60.5)	1.00		
	Yes	59(49.6)	60(51.4)	1.51(0.98–2.31)	1.03(0.54–1.94)	0.93
No of Comorbidities	1–2	117(43.2)	154(56.8)	1.00		
	> = 3	16(61.5)	10(38.5)	2.11(0.92–4.81)**	3.61(1.19–10.96)	**0.023**
No complications	1–2	91(44.8)	112(55.2)	1.00		
	> = 3	86(40.0)	129(60.0)	0.69(0.11–4.28)		
Antidiabetics	OHGA(s) alone	83(47.4)	92(52.6)	1.00		
	OHGA(s) + Insulin(s)	51(46.8)	58(53.2)	0.98(0.60–1.57)*	0.97(0.57–1.65)	0.920
	Insulin(s) alone	43(32.1)	91(67.9)	0.52(0.33–0.84)**	0.57(0.34–0.96)	**0.034**
Antihypertensives	Yes	91(44.8)	112(55.2)	1.00		
	No	86(40.0)	129(60.0)	1.22(0.83–1.80)		
Statins	Yes	83(39.0)	130(61.0)	1.00		
	Yes	93(45.8)	110(54.2)	1.32(0.91–1.99)*	0.77(0.36–1.64)	0.48
Aspirin	No	95(38.5)	152(61.5)	1.00		
	Yes	82(48.2)	88(51.8)	1.50(1.01–2.23)*	1.13(0.58–2.19)	0.74
No of medications	<5	100(39.1)	156(60.9)	1.00		
	> = 5	77(47.8)	84(52.2)	1.43(0.96–2.13)*	1.12(.56–2.25)	0.74
Source of drugs	For free	112(42.6)	151(57.4)	1.00		
	Paid	65(41.9)	90(58.1)	0.97(0.65–1.46)		
Adherence status	High (= 8)	52(27.4)	138(72.6)	1.00		
	Low(<6)	60(60.0)	40(40.0)	3.98(2.39–6.64)**	5.26(2.51–11.04)	**<0.001**

Percentages are calculated per row. Variables in bivariate analysis with p≤0.20 and ≤0.05 indicated by * and **, respectively. Statistically significant in multivariate analysis set at: p≤0.05, shown in **bold**. CI: Confidence interval at 95%; OHGA: Oral hypoglycemic agents; SMBG, self-monitoring of blood glucose.

#### Reported medication adherence

The mean (±SD) score of the MMAS in this study was 6.83±1.43. Accordingly, 45.7% of participants reported high adherence, 30.4% medium adherence and 23.9% low adherence. This shows that nearly one fourth of the total study participants were categorized under poor adherence. Moreover, patients with T2DM (25%) were more likely to be non-adherent to their medication than participants with T1DM (18%). Forgetfulness (33.7%) was the most prevalent reported reason for poor adherence followed by difficulty in remembering [sometimes] to take their medications (23.9%) and treatment plan inconvenience (22.7%).

#### Factors associated with poor medication adherence

Sex, Age, Education, Employment, Complication, Diuretics, Source of drug and ADEs were subjected for bivariate analysis. Subsequent multivariate logistic regression analysis revealed that female gender (AOR = 1.67, 95% CI:1.01–2.76, *p* = 0.046) and the presence of diabetes complication (AOR = 2.00, 95% CI:1. 20–3.32, *p* = 0.007) were positively associated with non-adherence, while participants who completed primary and secondary grade level of education (AOR = 0.42, 95%CI:0.18–0.96, *p* = 0.040 and AOR = 0.40, 95% CI:0.18–0.87, *p* = 0.021), respectively were a protective factor against the occurrence of non-adherence compared to participants with no formal education (Table 6).

**Table 6:** Bivariate and multivariate analysis of factors associated with medication non-adherence among ambulatory patients with diabetes on follow up at TASH, Addis Ababa, Ethiopia, 2017.

		Non-adherence		Odds Ratio		
Covariates	Categories	Yes	No	Crude(95% CI)	Adjusted(95%CI)	P-value
Sex	Male	39(19.8)	158(80.2)	1.00		
	Female	61(27.6)	160(72.4)	1.55(0.98–2.44)	1.67(1.01–2.76)	**0.046**
Age	Younger	63(21.2)	234(78.8)	1.00		
	Elderly	37(30.6)	84(69.4)	1.64(1.02–2.63)*	1.37(0.80–2.34)	0.250
Education	No formal education	21(40.1)	31(59.6)	1.00		
	Primary(1–8)	16(22.5)	55(77.5)	0.43(0.20–0.94)*	0.42(.18–0.96)	**0.040**
	Secondary(9–12)	24(19.8)	97(80.2)	0.37(0.18–0.74)*	0.40(0.18–0.87)	**0.021**
	College/University	39(22.4)	135(77.6)	0.43(0.22–0.82)*	0.60(0.27–1.37)	0.226
Employment	Employed	51(21.5)	186(78.5)	1.00		
	Unemployed	49(27.1)	132(72.9)	0.74(0.47–1.16)	.89(0.50–1.61)	0.702
Complication	No	61(20.4)	238(79.6)	1.00		
	Yes	39(32.8)	80(67.2)	0.53(0.33–0.85)*	2.00(1.20–3.32)	**0.007**
Diuretics	No	77(19.9)	275(78.1)	1.00		
	Yes	23(34.8)	43(65.2)	0.53(0.30–0.93)*	0.73(0.40–1.34)	0.31
Source of drug	Free	70(26.6)	193 (73.4)	1.00		
	Paid	30(19.4)	125(80.6)	0.66(0.41–1.07)	1.34(0.74–2.44)	0.33
ADEs	No	91(22.8)	308(77.2)	1.00		
	Yes	8(44.4)	10(55.6)	3.05(1.20–7.72)*	2.18(0.79–6.01)	0.131

Percentages are calculated per row.

*Variables in bivariate analysis ≤0.05,statistically significant set at: p≤0.05.

ADEs: Adverse Drug Events

### Treatment satisfaction

The Treatment Satisfaction with Medicines Questionnaire (SATMED-Q) is a brief, feasible, easy to self-administer, and multi-dimensional generic questionnaire comprising of 17 Likert-type items [24,27]. Around 90.67% (88.473–92.867) of the respondents said that undesirable side effects of the medications were interfering with their daily, leisure and physical activities. The total composite score (0–100) of satisfaction was 80.81% (72.23–89.39). However, with respect to medical care services, such as regarding detail information of the disease and drug treatment, only 54.37% (51.863–56.877) of participants were satisfied (Table 7).

**Table 7:** Treatment satisfaction in diabetes patients at diabetes clinic of TASH, 2017.

Items	Min	Max	Percent	Mean	SD
**Undesirable side effect**	3	12	**90.67**	**10.88**	**2.197**
Interference on physical activities	1	4	91.34	3.65	0.731
Interference on leisure activities	0	4	90.1	3.6	0.811
Interference on daily activities	1	4	90.49	3.62	0.778
**Treatment effectiveness**	1	12	**80.14**	**9.62**	**2.223**
Relieving symptoms.	0	4	78.59	3.14	0.899
Time to start working.	0	4	80.92	3.24	0.842
Feeling better	0	4	80.86	3.23	0.888
**Convenience of use**	1	12	**84.77**	**10.17**	**2.027**
Practical/actual of the medication	0	4	82.48	3.3	0.828
Easy to use/take the medication	0	4	86.24	3.45	0.833
Timetable taking of medication	0	4	85.77	3.43	0.901
**Impact on daily activities**	0	12	**84.91**	**10.19**	**2.482**
Impact on leisure and routine activities.	0	4	85.35	3.41	0.915
Impact on personal hygiene.	0	4	86.12	3.44	0.875
Impact on performing usual activities.	0	4	83.37	3.33	0.963
**Medical care**	0	8	**54.37**	**4.35**	**2.507**
Detail information of the disease	0	4	54.13	2.17	1.262
Detail information on drug treatment.	0	4	54.43	2.18	1.361
**General Satisfaction**	0	12	**81.16**	**9.74**	**2.013**
Desire to continue this treatment.	0	4	85.11	3.4	0.721
Comfortable with this treatment.	0	4	81.16	3.25	0.813
General satisfaction with this treatment.	0	4	77.57	3.1	0.79
**Total Composite score**	20	68	**80.81**	**54.95**	**8.58**

## Discussion

This study assessed DTPs, adherence and satisfaction of ambulatory patients visiting a tertiary care hospital, where chronic illnesses are managed. Prevalence of DTPs in the present study is in agreement with the previously conducted study in India [28]. However, it is slightly higher than the finding from Sri Lanka [14]. This slight variation could be due to the difference in study design (prospective) and DTPs classification system used (Pharmaceutical Care Network of Europe (PCNE) criteria) in the later study. In contrast, the finding of this study is considerably lower than studies conducted in Malaysia [9,10], Jordan [29] and Nigeria [12,30]. This discrepancy might be explained by variation in sample size and characteristics of the study participants. For example the Malaysian and the Nigerian studies included only T2DM with hypertension or dyslipidemia; the Jordanian study was conducted in five different teaching hospitals with relatively high number of study participants (n = 1494). Thus, the possibility of experiencing DTPs would be more in patients with T2DM as they had the chance to use poly drug therapies [2,13,31,32], and treatment for T2DM is more complicated than T1DM due to the fact that the body may produce enough insulin but not be able to use this insulin effectively in T2DM. T2DM usually occurs later in life, where multiple problems are apparent due to degeneration of organs [33,34].

Dosage too low (58, 28.0%) was the most commonly encountered DTP in the present study. This was similar with the study done in India [35]. In the current study, this problem was more commonly observed in type T1DM than T2DM patients (n = 11/61, 18% versus n = 47/357, 13.2%). The possible reason could be that majority of T1DM patients had been prescribed with Insulin and dose titration might be a difficult task for individual patients.

The use of ineffective drug therapy (26.1%) was the second most common DTP in this study. This finding was consistent with two studies conducted in India [35,36] and another study in China [37]. However, numerically, results in this study are higher than a number of findings in the literature [10,12,13,29,35–37] and lower than other studies in Sri Lanka [14] and the UK and Saudi [38].

Need additional drug therapy was the third most common DTP in this study, which accounted for 25.1%. This is in line with a study done in Sri Lanka [14], but relatively higher than reported from other studies [12,29,30]. On the other hand, unnecessary drug therapy, ADR and dose too high were less prevalent in the present study compared to other studies [12,14,29,30,36]. In a multination study done in the UK and Saudi [38], ADR and ineffective drug therapy were the most commonly identified DTPs. Nevertheless, the data is still concordant with a US study [13]. Drug classes found to be associated with DTPs in the current study such as antidiabetics (oral and insulin), antiplatelets and lipid lowering agents (statins) had also been implicated in the Malaysian studies (14, 17).

Multivariate logistic regression analysis (Table 5) revealed that seven variables were significantly associated with the occurrence of DTPs. Adjusted odds ratio (AOR) for sex (AOR = 2.31, 95% CI: 1.30–4.12, *p =* 0.004) indicated that females were more than two times likely to have a risk of developing DTPs than males (25.6% versus 16.7%). Furthermore, AOR for marital status (AOR = 2.58, 95%CI: 1.23–5.48, *p* = 0.013) indicated that ever married participants were 2.6 more likely to be associated with DTP compared to never married participants. This may be due to the fact that majority of the study participants were married. We have found no study in this regard to see the impact of this variable in DTPs.

AOR for place of residence (AOR = 0.30, 95%CI: 0.12–0.73, *p* = 0.008) revealed that participants residing outside of Addis Ababa were 70% less likely to be associated with DTPs. The occurrence of overall DTPs was substantially higher (5.6 fold) in patients with T2DM participants (95% CI: 1.21–26.04, *p* = 0.027). Meanwhile, medication adherence (AOR = 5.26, 95% CI: 2.51–11.04, *p*<0.001) indicated that participants with poor adherence to their medication had a strong positive relation with DTPs. Drug regimens, except insulin alone regimen (AOR = 0.57, 95%CI: 0.34–0.96, *p* = 0.034), were not statistically associated with DTPs. DTPs were substantially higher in participants on insulin alone treatment regimen compared to those participants on oral hypoglycemic agents (OHGAs) alone.

The number of co-morbidities per patient (≥3) was associated with a higher risk of developing at least one DTP, which was similar to studies conducted in UK and Saudi Arabia [39] and Wolaita Sodo, Ethiopia [40]. This could be explained by the fact that participants with more co-morbidities were more likely to have multiple medications, thus, they could be reluctant to take their medication appropriately. In addition, the risk of ADR could be increased in participants with comorbidities. In the current study, 7 out of 10 participants (70.6%) had different comorbid conditions and these might have impact on DTPs. This finding was in line with the study conducted by Claydon et al [39].

Female gender was significantly associated with developing DTPs. A good explanation for such issues was provided by WHO [6] and Lester [41], which reported more physical inactivity, obesity and overweight were observed in female than male. Moreover, this association might also be related with poor glycemic control, which was relatively more often seen in females (54%). Especially in developing countries, females are responsible for all family related issues and this might have impact on DTPs. This notion is in agreement with other studies showing few number of women achieve glycemic target compared to men [42–44]. Biological and psychosocial factors could also be responsible for gender differences in glycemic control [45,46]. Gender roles and power dynamics influence liability to diabetes, affect access to health services and health seeking behaviour of women, and magnify the impact of diabetes on them. Furthermore, many of women in the world had limited accesses to treatment, care and education [47].

Likewise, dosage too low was higher in female participants than male participants (67.2% versus 23.8%, p = 0.03). Other studies indicated that women need a higher insulin dose/kg than men [42]. Thus, prescribers should be aware of the need for titrating insulin dose, principally in women, to achieve the intended glycemic target in participants with poor glycemic control.

Many other **s**tudies have reported that increased age (>60 years old) could be considered as a risk factor for DTPs [10,35,36], but no statistically significant association was seen in the present study. This could be due to the difference in study participants’ average age (53 ± 13.6), which was less than in other studies participants’ average age. The difference could also be explained by the life expectancy variation between nations, which is less in Ethiopia, as well as by similar age group of the study participants, nearly half of them were between 40 and 60. Though, three in ten of participants had a history of hospitalization events due to hypoglycemic and/or hyperglycemic episodes, it failed to be significantly associated with DTPs, unlike other studies [40].

In the present study, 24% of the participants were non-adherent to their medications. This percentage was closer to a study conducted in India [48] and some studies in Ethiopia [49–51]. It was, however, lower compared to other studies conducted in Ethiopia [52–56] and higher than a study conducted in Uganda [57]. Factors including presence of diabetes complication, female gender, primary and secondary levels of education were demonstrated to be independently associated with level of adherence. Diabetes complications was demonstrated to be negatively associated with adherence, as non-adherent participants who developed diabetes complications were two-fold than those who didn’t develop diabetes complications, suggesting that complications could worsen non-adherence by 100%. This finding is in line with the study done in Jimma, Ethiopia [51].

Educational status was also shown to be negatively associated with non-adherence, as having primary or secondary level of education decreased the odds of participants’ being non-adherent to their medication by 58%. This association was consistent with studies conducted in Ethiopia [52, 53, 56] as well as in India [48]. The female gender was shown to be more likely to be non-adherent than the male gender. This association is also in agreement with the other studies [34,48]. Females in developing countries are responsible for the survival and continuation of life in their families and thus this burden could probably predispose them to be less-adherent to their treatment plan.

Unlike to the study reported by Wabe et al [58] and Kassahun et al [53], drug adverse events and diuretic medications were not significantly associated with adherence. Irrespective of their percentages, studies extensively reported that forgetting taking of medications, unavailability and cost of drugs, fear of drug side effects, regimen complexity, inconvenience of time schedule and inadequate provision of instruction as the most common reasons for non-adherence [48,50,52,53,55,58,59]. Surprisingly, there was no significant association with multiple medications regimen, drug related adverse effects and presence of co- morbid conditions. This was in contrary to reports from Teklay et al [51]. With regard to treatment satisfaction, the SATMED-Q has shown appropriate psychometric properties exploring patients’ satisfaction with treatment [27]. The overall treatment satisfaction was 80.81 (72.23–89.39), which was in line with the study done in Spain [24]. However, this finding is slightly higher than another study done by the same author [27].

Cross sectional study design by its nature does not allow determination of the temporal relationship between the dependent and independent variables. Some measures such as glycemic level could be underestimated or overestimated, given that the glucose monitoring parameter used was more of the values from their FBG/RBS measures (HgA1C was not used). Nevertheless, the higher sample size used in the present study could offset this limitation.

## Conclusion

A significant proportion of patients were satisfied with their treatment at TASH diabetic clinic. In addition to this, only one-fourth of the study participants were non-adherent to their treatment modalities. However, DTPs were considerably high among the study participants. The most common types of DTPs identified in the study were dosage too low, ineffective drug therapy and need additional drug therapy. The causes for the DTPs were use of sub-therapeutic dose, inappropriate drug selection and lack of adding synergistic or preventive drug therapy. Factors independently associated with developing DTPs were female gender, comorbid conditions, ever married, T2DM, and poor medication adherence. Hence, future interventions targeting prevention and resolution of DTPs deemed to be necessary.

## Supporting information

S1 File Participant information sheet, consent and data collection tools. (DOCX)

## Abbreviations

ADA/EASD: American Diabetes Association/European Association for the Study of Diabetes
ADEs: Adverse Drug Events
ADRs: Adverse Drug Reactions
AOR: Adjusted Odds Ratio
CI: Confidence Interval
CVD: Cardio Vascular Disease
DM: Diabetes Mellitus
DTPs: Drug Therapy Problems
eGFR: estimated Glomerular Filtration Rate
FBG: Fasting Blood Glucose
HbA_1c_: Glycosylated Hemoglobin A_1c_
IDF: International Diabetes Federation
LLAs: Lipid Lowering Agents
MMAS: Morisky’s Medication Adherence Scale
MTM: Medication Therapy Management
OHGA: Oral Hypoglycemic Agents
OR: Odds Ratio
PCNE: Pharmaceutical Care Network of Europe
SMBG: Self-Monitoring of Blood Glucose
SPSS: Statistical Package for Social Sciences
TASH: Tikur Anbessa Specialized Hospital
WHO: World Health Organization
