# Supplementary material for: Drug therapy problems, medication adherence and treatment satisfaction among diabetic patients on follow-up care at Tikur Anbessa Specialized Hospital, Addis Ababa, Ethiopia
Source: PLoS One. 2019 Oct 1;14(10):e0222985. doi: 10.1371/journal.pone.0222985 (PMC6772059; doi:10.1371/journal.pone.0222985)
Supplement: S1 File — (DOCX) [file pone.0222985.s001.docx]

Supporting information

## S1: Participant information sheet, consent and data collection tools

## Participants’ information sheet

Name of principal investigator: Professor Ephrem Engidawork

**Name of study area:** Endocrine and Metabolism unit of Tikur Anbessa Specialized Hospital

**Research budget covered by:** Addis Ababa University and the AAU MTM project

**Research objective:** To assess the magnitude and factors associated with drug therapy problems among ambulatory patients with diabetes at Tikur Anbessa Specialized Hospital.

**Significance of the study:** Drug therapy problems lead to poor clinical outcomes, increased healthcare costs and decreased quality of life; thus, they should be identified, resolved and prevented in a manner of the situation as much as possible.

**Risks:** The risk could be the lowest to the patient during the study. The only threat for the interviewee could be spending time (may be a maximum of 30 minutes was spent).

**Participant right:** The right of interviewees to withdraw at any time from the interview or not to participate and to escape questions which are not comfortable for them was reserved.

**Beneficial:** The study could be beneficial for patient’s quality service delivery system to come across and to promote an intervention for the future perspective.

**Confidentialities:** The study result did not include patient’s name and address. Hence confidentiality was kept as data was accessed only by ethically conducted, well trained and experienced health professionals of the study as well as interviewed and analyzed by the principal investigator.

**Agreement:** participants were fully voluntary to participate in the study.

**Whom to contact:** If participants have any kind of difficulties about the study, fell-free to contact the PI via the following address:-

Ephrem Engidawork (PhD)

Professor of Pharmacology
Dean, School of Pharmacy, 
College of Health Sciences, Addis Ababa University
Churchill Avenue
P.O. Box 1176
Addis Ababa, Ethiopia
e-mail: [ephrem.engidawork@aau.edu.et](mailto:ephrem.engidawork@aau.edu.et),  [ephrem.engidawork@gmail.com](mailto:ephrem.engidawork@gmail.com)

## Informed consent

**Name of the investigator**: Ephrem Engidawork

**Research title:** Drug Therapy Problems among Ambulatory Patients with Diabetes at Endocrine and metabolism Unit of Tikur Anbessa Specialized Hospital, Ethiopia

Card number: Code number__________

1. I, confirm that, I understand the information sheet for the above study and have had the
 opportunity to ask questions.

2. I, understand that my participation is completely voluntary, and that I am free to
 withdraw at any time, without giving any reason, without legal rights being affected.
3. I, understand that my response will be looked, and secured and necessary information will be extracted. I give you a permission to have an access to all my response.

4. I, agree to take part in the above study. I would like to confirm my agreement by signing.

Participant’s name _________________________ Signature:___________date_______
Data collector’s name:______________________Signature:___________date_______

**I thank you very much for your willingness to participate honestly and cooperatively!**

### Data collection tools (English version)

This tool was used to assess **“Drug Therapy Problems among Ambulatory Patients with Diabetes at Endocrine and Metabolism Unit of Tikur Anbessa Specialized Hospital”**

Dear data collector(s),

First of all I would like to thank you for your willingness to accept my request and to participate as a data collector in this study which will surely be of great significance in contributing to determine the magnitude and factors associated with drug therapy problems among ambulatory patients with diabetes on follow up at TASH. This data will then enable the concerned bodies to emphasize on the identified problems and the associated factors to formulate and implement Medication Therapy Management (MTM) services as a part of pharmaceutical care services in turn to identify, resolve and prevent them for the future. Finally, I would like to greatly acknowledge, in advance, your indispensible roles in maintaining the quality of this data honestly and effective output of the present study. I promise you to be timely respond whenever you face challenges during the data collection period.

**The principal investigator!**

**Greeting for the participants**

First of all, thank you for your willingness to participate honestly on this study.

My name is .I will like to interview you today on drug therapy problems and associated factors regarding your medical condition while you are undergone antidiabetic medications treatment. Confidentiality regarding this interview will be strictly maintained. At any time during our interview, please feel-free to let me ask if you have any questions or we can stop the interview at any time for any reason even you can escape any question which is(are) not-comfortable for you. I would like you to assure that there is no problem upon participating in this study except it may be spent your time (max of 30 minutes).

**I thank you very much for your time and willingness to answer this question honestly!**

**Patients’ Socio-demographic information**

| 1. **Socio-Demographic Characteristics:** | | | | | | | | |
| --- | --- | --- | --- | --- | --- | --- | --- | --- |
| 1. Age(in year): | | ____________________________ | | | | | | |
| 2. Sex | | Male | | | | Female | | Pregnant: Yes No |
| 3. Marital status: | | Single | | | | Married | | Divorced Widowed |
| 4. Religion | | 1. Orthodox | | | | 2. Muslim | | 3. Protestant 4. Others_____________ |
| 5. Educational status | | no formal education  Grade 1-8 | | | | Grade 9-10  Grade 10-12 | | College diploma  University degree and above |
| 6. Place of Residence | | Urban(Addis Ababa) Rural) our of Addis Ababa) | | | | | | |
| **7.** Occupation | | Government Retired No job(houswife)  Private office Self-Employed(farmer, daily laborer) | | | | | | |
| 1. Monthly income | | ________ETB | | | | | | |
| 1. Cigarette Smoker | | | | Yes No | | | Ex-smoker/ former smoker | |
| 1. Alcohol user | | | | Yes No | | | Ex-drinker/former drinker | |
| 11.Physical  Activity | Walk | | Yes | No , if yes, for how long? | | | <30minutes /day >30minutes/day | |
|  | Sport | | Yes | No if yes, for how long? | | | Daily 1-3d/week 4-6d /week | |
| **II. Clinical characteristics(supplementary to the information obtained from medical record chart):** | | | | | | | | |
| 1.Duration of diabetes | | | Years. 2. Duration of treatment______________years. | | | | | |
| 3.Hospitalization due to diabetes | | | Yes | No | If, yes due to Hyperglycemia Hypoglycemia  others, specify ______________________________ | | | |
| 4.Traditional/herbal medicine use/OTC | | | No Yes , If yes list them____________________________________ and  for what purpose___________________________________________________ | | | | | |
| 5.Use of Home SMBG | | | No Yes , | | | | | |
| 6.Source of medication | | | For free Paid | | | | | |

**Data abstraction format for chart review**

Card Number_______________ Unique ID/Code: ___________Age (in year) _____________

Weight (kg) _____________Height (cm) _________Body mass index (BMI)[kg/m2] ________

1. Relevant laboratory series results (Lab Findings of at least for three consecutive results).

| Parameters | Time of sequence | Most Recent | Visit_3_ | Visit_2_ | Visit_1_ | Average |
| --- | --- | --- | --- | --- | --- | --- |
| Blood glucose level and BP | FBS(mg/dL): |  |  |  |  |  |
|  | HbA1c (%): |  |  |  |  |  |
|  | BP, mmHg |  |  |  |  |  |
| Lipid profiles | LDL: mg/dl |  |  |  |  |  |
|  | HDL: mg/dl |  |  |  |  |  |
|  | TG: mg/dl |  |  |  |  |  |
|  | Total Cholesterol |  |  |  |  |  |
| OFTs | ALT/SGOPT |  |  |  |  |  |
|  | AST/SGPT |  |  |  |  |  |
|  | ALP |  |  |  |  |  |
|  | BUN |  |  |  |  |  |
|  | SrCr |  |  |  |  |  |
|  | eGFR |  |  |  |  |  |

1. Current medical conditions and medications (including Comorbid and complications) (in sequence)

| Medical Problem lists | Product name (Generic Name) | Regimens (dose, route, frequency, duration) | | | | Response |
| --- | --- | --- | --- | --- | --- | --- |
|  |  | Current | Visit_3_ | Visit_2_ | Visit_1_ |  |
|  |  |  |  |  |  |  |
|  |  |  |  |  |  |  |
|  |  |  |  |  |  |  |
|  |  |  |  |  |  |  |
|  |  |  |  |  |  |  |
|  |  |  |  |  |  |  |
|  |  |  |  |  |  |  |

1. Adverse Drug Events(documented):

| Drug Regimen | Adverse drug reaction /Drug allergies /Other alerts | Event date |
| --- | --- | --- |
|  |  |  |
|  |  |  |

1. Past medical and medication history (hospitalizations, surgical procedures, injuries, pregnancies). 1. Poorly GC 2. Sub-optimal GC 3. Fairly GC 4. Good/Well /optimal GC 5. Not stated

- Based on the most recent glucose level(FBS/RBS or A1c): (if same with the above, you can skip it)

1. Poorly /Sub-optimal controlled 2. Good/Well /optimal controlled 3. Cannot state

**Cipolle’s drug therapy problems classification system**

| **Domains** | **Drug Therapy Problems** | **Common Causes** | |
| --- | --- | --- | --- |
| i. Indication | Unnecessary drug therapy | | - No medical condition at that time - No need of drug for that condition |
|  |  | | - Duplicate therapy |
|  |  | | - Non-drug therapy indicated |
|  |  | | - Treating avoidable ADR |
|  | 1. Needs additional drug therapy | | - Addictive or Recreational drugs |
|  |  | | - Untreated indication |
|  |  | | - Preventive or prophylactic |
|  |  | | - Synergistic or potentiating |
| ii. Ineffectiveness | 1. Needs different drug product | | - Inappropriate drug selection |
|  |  | | - Condition refractory to drugs |
|  |  | | - Dosage form inappropriate |
|  |  | | - Not effective for the condition |
|  | 1. Dosage too low | | - Wrong dose(sub-therapeutic dose) |
|  |  | | - Frequency inappropriate |
|  |  | | - Drug interaction |
|  |  | | - Duration inappropriate |
| iii. Safety | 1. Adverse drug reaction | | - Undesirable effect not dose related |
|  |  | | - Unsafe drug for patient |
|  |  | | - Drug interaction not dose related |
|  |  | | - Allergic reactions |
|  |  | | - Contraindication present |
|  | 1. Dosage too high | | - Wrong dose(over therapeutic dose) |
|  |  | | - Frequency inappropriate |
|  |  | | - Duration inappropriate |
|  |  | | - Drug interaction |
|  |  | | - Incorrect administration |
| iv. Adherence | 7. Non-adherence | | - No willingness to take the drug |
|  |  | | - Patient forget to take the drug |
|  |  | | - Direction is not understood |
|  |  | | - Patient cannot swallow/administer |
|  |  | | - Cost of medication too expensive - Unavailability of medication - Disbelieves on the drug effectiveness - Patient felt better or worse - Fear of adverse events - Regimen complexity |

Total number of identified DTPs =________ Total number of identified Causes_________

**Logical questions to identify whether or not the patient is experiencing a drug therapy problem**

1. Is the medication (indication) appropriate?
   - 1. Is there a clinical indication for each medication being taken?
     2. Are all of the patient's medical conditions that can benefit from drug therapy being treated?
2. Is the drug therapy effective for the disease condition?
3. Is the most effective drug product being used?
4. Is the dosage of the medication sufficient to achieve the goals of therapy?
5. Is the dosage form appropriate?
6. Is the drug therapy as safe as possible?
   1. Is there any adverse drug reaction being experienced?
   2. Is there any sign of toxicity (based on clinical parameters (signs and symptoms) or lab. values)?
7. Is the patient adherent to his/her medication?
8. Is the patient willing and able to take the medications as intended?
9. Adherence assessment

- About how many days of the month have you missed taking prescribed medications?
- Have you ever stopped or started taking any of the prescribed medications on your own?
- Have you had difficulty taking your medications as prescribed? If so, why?
  - Is it because;(Reasons):
    - 1. You do not understand the instructions?
      2. You prefer not to take the medication?
      3. You forget to take the medication?
      4. The drug product is too expensive for you?
      5. You experienced side effects while taking the medications?
      6. You cannot swallow or self-administer the drug product appropriately?
      7. The drug product is not available for you?
      8. Others, please specify.______________________________

**Table 4: 8-Items Morisky Medication Adherence Scale- (MMAS-8)**

| **No** | **Items** | | | | **No** | **Yes** |
| --- | --- | --- | --- | --- | --- | --- |
| **1** | Do you sometimes forget to take your pills? | | | | +1 | 0 |
| **2** | People sometimes miss taking their medications for reasons other than forgetting. Thinking over the past two weeks, were there any days when you did not take your medicine? | | | | +1 | 0 |
| **3** | Have you ever cut back or stopped taking your medicine without telling your doctor because you felt worse when you took it? | | | | +1 | 0 |
| **4** | When you travel or leave home, do you sometimes forget to bring along your medicine? | | | | +1 | 0 |
| **5** | Did you take all your medicine yesterday?[reverted] | | | | 0 | +1 |
| **6** | When you feel like your symptoms are under control, do you sometimes stop taking your medicine? | | | | +1 | 0 |
| **7** | Taking medicine every day is a real inconvenience for some people. Do you ever feel hassled about sticking to your treatment plan? | | | | +1 | 0 |
| **8** | How often do you have difficulty remembering to take all your medicine? | | | |  |  |
|  |  | 1. Never/Rarely | A=4 | **The item result** ÷ **4=**  1.00  0.75  0.50  0.25  0.00, respectively | + |  |
|  |  | 1. Once in a while | B=3 |  |  |  |
|  |  | 1. Sometimes | C=2 |  |  |  |
|  |  | 1. Usually | D=1 |  |  |  |
|  |  | 1. All the time | E=0 |  |  |  |
|  | **Total score** | | | |  |  |

**Scoring scale**:

Each ‘no’ response rated as 1 and each ‘yes’ response rated as 0 for items 1 to 7, except for item 5 reversed. For item 8, the code (0-4) has standardized by dividing the result by 4 to calculate the summated score. The MMAS-8 range from 0 to 8 with the total score of **<6= poor adherence, 6-<8= medium adherence, 8= high adherence).**

**Reasons for poor medication Adherence**:

Mark × No √ Yes

- Cost of medication too expensive
- Inadequate availability of medication
- Fear of medication adverse events
- Regimen complexity
- Difficulty of administration
- Inadequate instruction
- Simply Forgetfulness
- Disbelief in drug effectiveness
- Patient prefers not to take
- Feeling better or worse
- Due to work load/busy
- During fasting
- Others______________________

**Assessment of adverse drug reaction/allergic reaction**

1. Have you experienced any undesirable, unusual adverse drug events /allergic reaction to the prescribed medicines for you since the last 1 year? Yes No:

If yes, describe the manifestation of the events_____________________

1. If your answer is “yes” for question number-1, answer the following questions:
   1. Did the adverse event occur after the suspected drug was administered?

Yes No Don’t know

- 1. Did the adverse reaction improve when the drug was discontinued or a specific antagonist/antidote was administered? Yes No Don’t know
  2. Did the adverse reaction reappear when the drug was re-administered?

Yes NoDon’t know

- 1. Are there alternative causes (other than the drug) that could have on their own caused the reaction? Yes: No Don’t know
  2. Was the reaction more severe when the dose was increased or less severe when the dose was decreased? Yes: No Don’t know
  3. Did you have a similar reaction to the same or similar drugs in *any* previous exposure?

Yes: No: Don’t know

**Key informant questionnaire (Self-administered**)

**Physicians’ perspective in pharmacological management of diabetes patients**

Dear all!

First of all, thank you for your willingness to answer this questionnaire. Currently, we are working a project research on title “drug therapy problems and potential associated factors” regarding physicians’ experience and existing practice while managing adult ambulatory diabetic patients. The purpose of this study is to identify associated factors and then to empower the management of patients in diabetes clinic of Tikur Anbessa Specialized Hospital.

1. Current level of education (position)?
2. Sub-specialist/senior(Internist, Endocrinologist, Diabetelogist)
3. Fellows
4. Resident(R1, R2 or R3)
5. Other, specify_____________________________________________
6. Which type of guideline(s) you are using for pharmacological management of your patient?
7. Institutional based 3. National based/STG

2. International based 4. Mixed 5. Not using any of the above

1. Specifically, what international guideline(s) you are using?
2. IDF 2. ADA 3. ADA-EASD 4. Others, Specify;_________________
3. Do you follow the guideline strictly? (keeping individualizing patients) 1. Yes 2. No
4. What could be the possible reasons (justifications/obstacles) for the development of DTPs (if any)? Or that can hinder to follow the guideline?
5. Patient preference (Eg. resistant to initiate insulin or to add dual therapy)
6. Adherence issue (Eg. falling the patient not to take medication as prescribed)
7. Medication availability issue (Eg prescribing Glibenclamide instead of metformin)
8. Side effect of drugs (Eg. GI intolerance of Metformin, or Injection site pain/insulin)
9. Cost issue (Eg. adding of Glibenclamide to metformin instead of glimepiride or other)
10. Poor accessibility of the guidelines (Eg. using hardcopy as a desk-reference)
11. Patient load (number of patient vs physician, this may hinder provision of counseling)
12. Health facility/administration issue (Eg. Limitation of rooms for examination)
13. Others, specify_________________________________________________________

6. What monitoring parameter commonly you are using for the evaluation of glycemic control?

1. A1c 2. FBS 3. RBS 4. Others, specify________________

7. Generally, what could be the possible barriers for diabetes treatment within this center?

1. Patient related (specify if any) ___________________________________________

2. Professionals related (specify if any) ______________________________________

3. Institution related (specify if any) ________________________________________

4. Others (specify if any) __________________________________________________

**I thank you very much for your time and willingness to be filled this data honestly!**

# የጥናት መረጃ መጠይቅ በአማርኛ (Amharic/Ethiopic version)

**በጥናቱለሚሳተፉ የፍቃደኝነት ማረጋገጫ**

እኔ ለዚህ ጥናት ሙሉ በሙሉ ተነግሮኝ ተረድቻለሁ።አላማውም በተመላላሽ የስኳር ህመምተኞች ህክምና ላይ ያሉ ከመድኃኒት የተያያዙ ችግሮች መገምገም እና መለየት ነው።ይህ ተሳትፎ በፈቃደኝነት ላይ የተመሰረተ መሆኑን ተረድቻለሁ። በተጨማሪም የተረዳሁት በዚህ ተሳትፎ የአገኗኛለሁ የምለው ምንም ዓይነት የተለየ አገልግሎት፤ክፍያ ወይም ስጦታ እንደማይኖር ተረድቻለሁ። ይህ ውል የሚያገለግለው ለዚህ ጥናት ብቻ ነው።ከዚህ በታች ስሜ የተገለፀው በዚህ ጥናት ለመሳተፍ ተስማምቼለሁ።

ስምፊርማቀን

1. ተሳታፊ፤_________________ __________________ ____________________
2. መረጃ ሰብሳቢ፤_________________ _______________ ___________________
3. የጥናቱ ዋና ተመራማሪ ፤ ፕሮፌሰር ኤፍሬም እንግዳወርቅ _________________ _________________

ለተሳትፎዎ አመሰግናለሁ!

**ሀ. የታካሚዎች ማህበረሰባዊ ባህርያቶች መረጃ መሰብሰብያ ቅጽ**

- [በተዘጋጀለትሳጥንየ√ ምልክትያድርጉ] ካርድቁ*______________________*
- በመጀመርያበጥቁርአንበሳየስኳርህመምተኛሁኖመድኃኒትከጀመሩከ 3 ወርበላይመሆናቸውያረጋግጡ

| 1.እድሜ፤________ | | 2.ፆታ፤ | | | | | | | | ወንድ ሴት | | | | | | | | እርጉዝ: አዎአይደለም | | |
| --- | --- | --- | --- | --- | --- | --- | --- | --- | --- | --- | --- | --- | --- | --- | --- | --- | --- | --- | --- | --- |
| 3.የጋብቻሁኔታ፤ | | ያላገባ/ች | | | | | | | | ያገባ/ች | | | | | | | | አግብቶ/ታየፈታ/ች ሚስቱ/በሏየሞተችበት/ባት | | |
| 4. እምነት | | ኦርቶዶክስ | | | | | | | | ሙሰሊም | | | | | ፐሮቴስታንት ካቶሊክሌሎች | | | | | |
| 5.የትምህርትሁኔታ፤ | | መጻፍ ና ማንብብ አይችሉም  መጻፍና ማንበብይ ችላሉ  አንደኛ ደረጃ (1^ኛ^-8^ኛ^) | | | | | | | | | | | | | | | | ሁለተኛ ደረጃ (9^ኛ^-12^ኛ^)  ኮሌጅ ዲፕሎማ  ዩኒቨርስቲ ዲግሪ እና ከዛበላይ | | |
| 6.አሁንየሚኖሩበት | | | | | | ከተማ[አ . አ ] ገጠር [ ከአ . አውጭ/ክፍለሀገር]______________________ | | | | | | | | | | | | | | |
| 7. የሥራሁኔታ፤ ቋሚ የመንግስት መስርያ ቤት ሰራተኛ ጡረታ ሥራ የሌለው/የሌላት  ቋሚ የግል መስርያ ቤት ሰራተኛ የግልስራያለው/ያላት | | | | | | | | | | | | | | | | | | | | |
| 8. በወርሚያገኙትገቢ_________________________________(የኢትዮጵያብር) ገቢየሌለው/የሌላት | | | | | | | | | | | | | | | | | | | | |
| 10. ሲጋራ ያጨሳሉ? | | | አላጭስም አዎ | | | | | | | | | | ድሮ አጭስ ነበር አሁን ግን አቁሜለሁ | | | | | | | |
| መልስዎ አዎ ከሆነ፤መጠኑበቀን________________ፓኬት | | | | | | | | | | | | | | | | | ለስንትዓመት_______________________________ | | | |
| 11. መጠጥ (አልኮል) ይጠጣሉ? | | | | | | | አልጠጣም አዎ | | | | | | | | | | ድሮ እጠጣ ነበር አሁን ግን አቁሜለሁ | | | |
| መልስዎአዎከሆነ፤መጠኑበቀን__________________ | | | | | | | | | | | | | | | | | ለስንትዓመት_______________________________ | | | |
| 12. ቡና ይጠጣሉ? አልጠጣም አዎ  መልስዎአዎከሆነ፤መጠኑበቀን__________________ሲኒ በሱኳር ያለሱኳር | | | | | | | | | | | | | | | | | | | | |
| 14. የአካልእንቅስቃሴ | የእግርጉዞያዘወትራሉ? | | | አላደርግም  አዎ | | | | | | | መልስዎአዎከሆነበቀንለምንያህልግዜይጓዛሉ? | | | | | | | | ከ30 ደቂቃበላይ  0  ከ 30 ደቂቃበታች | |
|  | የአካልብቃት/ ስፖርትያዘወትራሉ? | | | | | | | አላደርግም  አዎ | | | | መልስዎ አዎ ከሆነ በሳምንት ለምን ያህል ግዜ ይሰራሉ? | | | | | | | | በየቀኑ / ሣምነትሙሉ  3-6ቀናት 1-2ቀናት |
| 15.በስኳርበሽታዎትምክንያትሆስፒታልተኝቶያውቃሉ? | | | | | | | | | | | | | | አላውቅም አዎ | | | | | | |
| መልስዎትአዎከሆነምክንያትዎ | | | | | | | | | የደም ስኳር መጨመር = በዓመትስንትግዜ?______________________የደምስኳርማነስ = በዓመትሰንትግዜ?_________________________ | | | | | | | | | | | |
| 16. የባህል እና ያለ ሓኪም ትእዛዝ መድኃኒት ይወስዳሉ? | | | | | | | | | | | | | | በፍጹም አዎ፤ አዎ ከሆነ ይገለፅ________________ | | | | | | |
| 19. የመድኃኒትዎ ምንጭ | | | | | ሁሉ ግዜ በነፃ አልፎ አልፎ በነፃ ሁሉ ግዜ በገንዘብ አልፎ አልፎ በገንዘብ | | | | | | | | | | | | | | | |
| 17.በሽታዎት ተመርምሮ ካወቁ ስንት ግዜ ሆኖቷል ?_________የስኳር መድኃኒት መውሰድ ከጀመሩ ስንት ግዜ ሆኖቷል ?_______ | | | | | | | | | | | | | | | | | | | | |
| 20. በቤትዎየሱኳር (Glucometere) መሳርያይጠቀማሉ? | | | | | | | | | | | | | | | | አዎ አይደለም/የለኝም | | | | |

**ለ. የስኳርበሽታታካሚዎችበመድኃኒትአወሳሰድ/አጠቃቀምዙርያግምገማበተመለከተ(ሞሪስኪስምንት)**

| **ተቁ** | **ጥያቄዎች** | | **አይደለም** | **አዎ** |
| --- | --- | --- | --- | --- |
| **1** | አንድ አንዴ መድኃኒት መውሰድ ረስተው ያውቃሉ? | | - **+1** | - **0** |
| **2** | አንድ አንድ ሰዎች ከመርሳት ውጭመድኃኒትዎ ያለመውሰድችግር አለ፤  ባለፈው ሳምንት ውስጥ መድኃኒትዎ ያልወሰዱበት ቀን አለ? | | - **+1** | - **0** |
| **3** | ሃኪምዎትን ሳያማክሩ በበሽታዎ ብሶት ምክንያት መድኃኒትዎ መውሰድ አቋርጠው ያወቃሉ? | | - **+1** | - **0** |
| **4** | ወደ ሌላ ቦታ ሲጓዙ ወይንም ከቤት ወጥተው ሲሄዱ መድኃኒትዎ ይዘው ከመሄድ ረስተውት ያውቃሉ? | | - **+1** | - **0** |
| **5** | በትላንትናው ዕለት ሁሉንም መድኃኒትዎ በትክክል ወስደዋል? | | - **0** | - **1** |
| **6** | የበሽታዎ ምልክቶች አልቀነሰም ብለው አንድ አንዴ መድኃኒት መውሰድ አቋርጠው ያውቃሉ? | | - **+1** | - **0** |
| **7** | መድኃኒት በትክክል አለመውሰድ የአንድ አንድ ሰዎች ዋነኛ ችግር ነው፤  የህክምና መርሃ ግብርዎ በትክክል መከታተል ችግር ሆኖበት ያቃል? | | - **+1** | - **0** |
| **8** | ሁሉኑም መድኃኒት መውሰድዎን ማስታወስ የከበድዎት ስንት ግዜ ነው?   1. ፈጽሞ🖵 2. በሆነ ግዜ አንድ ግዜ🖵 3. አንድ አንድ ግዜ🖵 4. አብዛኛው ግዜ🖵 5. ሁሉ ግዜ🖵 | 1. 4 2. 3 3. 2 4. 1 5. 0   የመልስ ውጤት በ 4 ሲካፈል የሆናል= |  |  |
|  | **ድምር** | |  |  |

- **ለመድኃኒትዎበአግባብአለመውሰድምክንያትሊሆኑየሚችሉ(ከታችከተዘረዘሩትከአንድበላይመምረጥይቻላል)**

1. የመድኃኒት ዋጋ ውድነት -----------------------------------------------------------አዎአይደለም
2. የመድኃኒት በቂ አቅርቦት አለመኖር-------------------------------------------------አዎአይደለም
3. የመድኃኒቱ የጎንዮሽ ጉዳት ፍርሀት--------------------------------------------------አዎአይደለም
4. የመድኃኒቱ አወሳሰድ ዘዴ ከባድነት እና ብዙ ዓይነት መድኃኒት በመሆኑ--------------አዎአይደለም
5. የመድኃኒቱ አወሳሰድ የግዜ ሰሌዳ አለመመቻቸት------------------------------------አዎአይደለም
6. ስለበሽታው እና መድኃኒቱ በቂ የሆነ የምክር አልግሎት አለማግኘት------------------አዎአይደለም
7. ያለምንም ምክንያት በመርሳት ብቻ --------------------------------------------አዎአይደለም
8. የመድኃኒት ፈዋሽነት አለመተማመን/አለማመን -------------------------------------አዎአይደለም
9. መድኃኒት ላለመውሰድ መምረጥ---------------------------------------------------አዎአይደለም
10. ህመሙ ሲሻሎት ወይም ሲብስቦት-------------------------------------------------አዎአይደለም
11. በስራ ጫና ምክንያት የሚወሰድበት ሰዓት ማሳለፍ እናመተው-----------------------አዎአይደለም
12. በፆም ምክንያት ሰዓቱን ጠብቆ አለመውሰድ----------------------------------------------አዎአይደለም
13. ሌሎች_________________________________________________________________________

**መ.ከመድኃኒትየተያያዙየጎንዮሽጉዳትወይምየሰውነትቁጣግምገማበተመለከተ**

1. ከባለፈው ዓመት ወዲህ ያለው ግዜ ከሚወስዱባቸው መድኃኒት በተያየዘ ያልተለመደ/ያልተፈለገ ሁኔታ/የጎንዮሽ ጉዳት/ የሰውነት መቆጣት አጋጥሞት ያውቃል? አዎ አይደለም፤ መልስዎ አዎ ከሆነ የመድኃኒቱ ዓይነት እናየሁኔታው ምልክት ይግለጽ፡­­__________________________________________________________________________
2. የጥያቄ ቁጥር 1 መልስዎ አዎ ከሆነ፤ለሚከተሉትን ጥያቄዎች መልስ ይስጡ
   1. ጉዳቱ/ቁጣው መድሐኒቱን ከወሰዱ በኋላ ነው የተከሰተው?-------------------------አዎአይደለምአይታወቅም
   2. መድኃኒቱ ካቋረጡት በኋላ ጉዳቱ አሻሽሏል/ቁሟል? --------------------------------አዎአይደለምአይታወቅም
   3. መድኃኒቱ እንደገና ሲወስዱት ምልክቱ/ጉዳቱ እንደገና ተከስቷል? --------------------አዎአይደለምአይታወቅም
   4. ከመድኃኒቱ ውጭ ሌላ እንደዚ ህዓይነት ጉዳት/ቁጣ ሊያመጣ የሚችል አለ?-----------አዎአይደለምአይታወቅም
   5. ጉዳቱ የመድኃኒቱ መጠን ሲጨምር ብሷል ወይም መጠኑ ሲቀንስ ቀንሷል? -----------አዎአይደለምአይታወቅ
   6. ከዚህ በፈት እንደዚህ ዓይነት ተመሳሳይ ጉዳት ለተመሳሳይ መድኃኒት አጋጥሞት ነበር?-አዎአይደለምአይታወቅም
